# Tailoring the continuum of care for substance use problems to persons with an Islamic migration background: a co-creative case study

**DOI:** 10.3389/fpsyt.2023.1205362

**Published:** 2023-11-24

**Authors:** Aline Pouille, Arafat Bouachiba, Clara De Ruysscher, Freya Vander Laenen, Wouter Vanderplasschen

**Affiliations:** ^1^Department of Special Needs Education, Research Group Recovery and Addiction Ghent University, Ghent, Belgium; ^2^De Cocon (Youth Care Service) and Drugstories (Organization for Drug Prevention Activities), Ghent, Belgium; ^3^Department of Criminology, Penal Law and Social Law, Institute for International Research on Criminal Policy, Ghent University, Ghent, Belgium

**Keywords:** co-creative research, addiction, recovery, substance use treatment, Muslim, migrant, minorities

## Abstract

**Introduction:**

Structural inequity and stigma impose barriers toward substance use prevention and recovery support for persons with an Islamic migration background in non-Islamic majority countries. Similar issues of differential power often keep them silenced in research. Therefore, we explore the continuum of care for substance use problems regarding persons with an Islamic migration background.

**Methods:**

We draw from a co-creative case study with Arafat, whose lived and professional experiences as a Muslim with a history of problem substance working in the field, were blended with academic literature through the process of ‘plugging in’.

**Results:**

We discuss (1) culturally competent and selective substance use prevention, (2) facilitating access to adequate support services, (3) culturally competent substance use treatment and (4) supporting long-term recovery for persons with an Islamic background from a combined academic, professional and lived experiences perspective.

**Discussion:**

We discuss the need for tailored interventions that are able to overcome structural inequities and address ethnocultural sensitivities, needs and strengths. Intermediary community organizations, cultural competence of treatment and recovery-oriented systems of care may bridge the gaps between what is needed and what is available. However, it is important to be conscious that hands-on solutions at the personal level do not absolve the responsibility of searching for systemic solutions. Furthermore, awareness of the fine line between cultural competence and culturalization, taking into account the danger of essentializing, othering and overlooking other intersectional traits of diversity, is needed.

## 1 Introduction

Worldwide, more than 60 million persons with a migration background are of Islamic-majority country descent. There has been substantial migration of persons from Islamic-majority countries to Europe, shaped by Europe’s colonial history, as well as policies encouraging labor migration after World War II (1, 2). This is also reflected in the Belgian population, where persons with Islamic migration backgrounds^1^ make up the largest ethnocultural minority population, meaning that they have a shared ethnic background of Islamic-majority countries and a shared cultural background related to their religion that differs from the majority population (3–6).

While Islam condemns non-medical psychoactive substance use as ‘haram’ or forbidden, (problem) substance use does occur within Muslim communities (7, 8). A recent systematic review comparing substance use among migrant and native adolescents in Europe has shown that, in comparison to native-born adolescents, alcohol consumption is lower among Islamic migrants, cannabis consumption is similar or lower, and the use of tobacco and illicit drugs other than cannabis show mixed results (9). Lower alcohol consumption among persons with Islamic migration backgrounds is congruent with alcohol use reported in Islamic-majority countries and may be related to the explicit mention of alcohol in the Quran (10). Other psychoactive substances are condemned more implicitly, which may explain the weaker correlation between religiousness and other psychoactive drug use compared to alcohol use (9, 10). Yet, research regarding substance use prevalence among Islamic migrant populations is scarce and numbers might be distorted due to stigma- and taboo-related underreporting (7).

To prevent problem substance use and support recovery,^2^ scholars have called for the application of a continuum approach (12). This holistic view on addressing problem substance use and supporting recovery on a continuum of care has also been embraced by the Flemish government, as articulated in a Vision Note of 2015 (13). The continuum of care ranges from the prevention of problem substance use among individuals at increased risk to secondary prevention and early intervention. It extends further to harm reduction, outpatient and inpatient substance use treatment, relapse prevention, and long-term recovery maintenance (14, 15). While (aggravation of) substance use problems is prevented by increasing protective factors and decreasing risk factors, recovery may be supported through increasing personal, social and community recovery capital (i.e., resources that initiate, facilitate and sustain substance use recovery) (16, 17). Risk and protective factors are intersectionally related and spread across the macro-to-micro continuum, resulting in differential influences on substance use outcomes (18, 19). They may also differentially impact recovery of problem substance use among persons with an Islamic background, through the mediating impact of recovery capital (17). Recovery capital is unequally distributed across populations, with migrant and ethnic minority populations being especially disadvantaged due to socio-economic and health disparities (20).

For persons with an Islamic migration background, active religious participation, a strong sense of ethnic identity (i.e., adopting intergenerationally transferred aspects of ethnic culture as a part of one’s identity, such as norms, values and behavioral characteristics) and a supportive social network have been identified as protective factors against problem substance use (21–23). However, persons with an Islamic migration background may also be confronted with several direct and indirect risk factors, often stemming from social exclusion and structural inequities. These risk factors include higher unemployment rates, limited educational opportunities, and experiences of stigma and discrimination (7, 24, 25). Stigma arises from the intricate interplay of stereotypes, prejudice, and subsequent discrimination, with religion, race, and ethnicity, as well as problem substance use as significant sources (26–28). Stigma is context-dependent and can vary between cultures (29). In that regard, research has pointed to double, and even triple stigma, indicating that persons with an Islamic migration background can be stigmatized due to (problem) substance use within both their own communities and broader society, as well as due to religion and ethnicity within society (30, 31). Research has also referred to possible accumulations of these and additional forms of stigma and their potential to have far-reaching consequences on the lives of those affected (27, 28). Recurrent stigma may lead to structural inequities, and even inequities, which are described as systematic disparities between groups with different levels of underlying social (dis)advantages that are not only unnecessary, but also unfair and unjust (32, 33). For instance, individuals with an Islamic background in France are significantly less likely to receive a job interview compared to their Christian counterparts. This has been linked with negative perceptions toward persons with an Islamic background due to fear-centered associations with terrorism and increasing right-wing political tendencies across Europe (19, 34–36). On a personal level, stigma may lead to self-stigma and identity disruption (37). Additionally, acculturation difficulties and ethnic conformity pressure may be stressors that form risk factors for substance use among persons with an Islamic migration background (38).

Muslim communities can have both a helping and hindering impact on recovery processes, dynamically increasing and decreasing access to recovery capital for persons with an Islamic migration background (31). They have the potential to offer spirituality and faith, a religious social network, a supportive community and an environment with restricted availability of substances as recovery resources, but at the same time, the moral load of substance use problems in Islam may keep people from disclosing substance use problems or seeking treatment and may lead to isolation and loss of social network (8, 10, 24, 39). Besides stigma, barriers to effective treatment include language barriers and knowledge gaps (knowledge of the treatment system among persons with an Islamic background and knowledge gaps regarding Islam among service providers), as well as a lack of religiously and culturally tailored interventions (39, 40).

Even though we have established that persons with an Islamic background may experience specific strengths and challenges related to problem substance use and recovery, research that explores how the continuum of care may be tailored to the needs of persons with an Islamic migration background in a non-Islamic majority country is currently lacking (22, 24). This research, therefore, explores the following research questions:

1. What challenges do persons with an Islamic migration background in a non-Islamic majority country with substance use problems face on the continuum of care?
2. Which interventions and resources may improve the continuum of care for persons with an Islamic background in a non-Islamic majority country?

We explore possible answers to these research questions through a co-creative case study that combines academic, personal and professional experience as supplementary ways of knowing. By crystallizing these perspectives, this research aims to gain a comprehensive understanding of the challenges that persons with an Islamic migration background may face across the interrelated aspects of the continuum of care for substance use problems as well as related needs and good practices of how to overcome these challenges and answer to these needs.

Persons with Islamic migration backgrounds who use substances are at risk of being marginalized because of culturally and historically rooted intersectional stigma related to both axes of oppression and within several ecological layers of society (24, 28). These unequal power relations also permeate research, mainly consisting of monological research *about* people with lived experiences rather than dialogical research together *with* people with lived experiences (41, 42). For science to remain true to its goal of improving people’s lives, it must ensure that those who are affected by research have a crucial role in shaping the meaning and generating knowledge during the research process (43, 44). In-depth and qualitative research methods that centralize the lived experiences of persons with first-hand experience are appropriate in this regard (7, 45, 46). Therefore, we engaged in a co-creative case study with a person whose voice contains a multiplicity of meanings due to both lived and professional experiences along the continuum of care. Yet, these experiences do not stand in a void. Instead of inhibiting the inclusion of ‘previously thought data’ in the research process, as is often the case in more traditional qualitative research designs, we ‘plug in’ existing knowledge from the mind of a researcher into these experiences, enabling to overcome so-called ‘limitations of voice’ and allowing multiple forms of knowledge to constitute one another and comprehensively explore the topic from a combined academic, professional and lived experiences perspective (47).

## 2 Methods

### 2.1 A co-creative case study

This co-creative case study took place in Ghent, a middle-size superdiverse city in Flanders (the Dutch-speaking part of Belgium), where approximately 37% of the inhabitants have a migration background, the majority with Islamic-majority country roots (48).

Co-creative research is an inclusive, participatory approach to research in which both the process and outcome aim for knowledge creation and social change, especially for those who are most likely to be affected by the work. It is characterized by collaboration between actors with diverse perspectives and expertise, which can synergize multiple epistemologies, sources of knowledge, ways of knowing, and worldviews (49). We follow Apers and colleagues (50), who point to co-creative research as suitable for untangling complex issues in migration studies and argue that co-creative research practices are more encompassing than other participatory methodologies. Essentially, the co-creative approach that we apply in this study is described by Tomlinson and De Ruysscher (51) in the following way:

*“Co-creation is much more far-reaching than simply involving the perspectives of persons with lived experience in research (e.g. as data source, executor of research tasks). Instead, conducting research in dialogical ways implies an intensive, non-linear and relational process that takes place between persons with lived experience and academics. A dialogical research process is one of continuous interaction and bricolaging together in all phases of the research trajectory; of searching for the most relevant research questions, of seeking out adequate research methods, of keeping an unfinalizable dialogue going about emerging insights and ideas.”* (p. 1284)

A case study has been defined as an intensive investigation of a person, a group of people or a unit (52). Case studies are fit to get an in-depth and holistic understanding of complex phenomena within a person, community or other bounded system by thoroughly diving into comprehensive and multilayered data in a certain context (53). However, as discussed in the introduction, rather than doing a case study *about,* this case study is co-created *with* a person who has lived and professional experiences as a person with an Islamic background and substance use problems within the continuum of care. By applying a co-creative case study approach, we aim to tackle epistemological inequalities and to democratize knowledge and knowledge production (11, 51, 54). Other studies that have applied a comparable methodology can be found in De Ruysscher and colleagues (46) and Moernaut et al. (55).

The study focuses on the challenges, needs, and good practices experienced by individuals with an Islamic migration background in a non-Islamic majority country, starting from the experiences of Arafat and broadening these to other contexts by including the academic knowledge of Aline in the research process and outcomes. This assemblage of academic, professional and experiential knowledge is also known as ‘plugging in’ (47). By plugging in, academic researchers go beyond the illusion of objectivity in qualitative data analysis. Instead, they bring in their own ideas that are shaped by what they read, wrote, and heard throughout their academic career. Through this assemblage of information, science, practice and personal experiences meet, interact and develop into a comprehensive and multilayered understanding of the challenges, needs and good practices on the continuum of care for persons with an Islamic migration background with substance use problems in a non-Islamic majority country (45, 47).

Aline, the first author of this paper, is a 29-year-old white woman of Belgian origin. For the last five years, she has been working as a PhD researcher at the Faculty of Psychology and Educational Sciences of Ghent University (Belgium). Her PhD research, which is coming to an end at the time of writing, concerns substance use recovery processes of persons with a migrant and ethnic minority background from a qualitative and lived experiences point of view. Throughout the years, she has gained an understanding of this topic by reading relevant scientific and grey literature, looking into policy documents, conducting interviews with persons with a migration background and ethnic minorities and substance use problems, relevant professionals and policy members and participating in thematically linked meetings with professionals. She has personal experience with substance use recovery in her family and has had a taste of migration experiences when her father migrated to another country in her youth and during a three-month international internship. This co-creative case study forms the culmination of her PhD research, allowing her to integrate previously encountered data and literature in the research process in order to come to higher levels of understanding strengthened by the lived and professional experiences of Arafat. Aline’s research is part of an on-going field of Belgian pedagogical and criminological research on substance use problems and mental health care in persons with a migration background, focusing on the patterns of substance use (56), equitable substance use treatment (57), cultural differences in help-seeking processes (58), cultural competence (59), identity constructions (60), among other topics. Aline’s current work is strongly built on and inspired by this line of research, both theoretically and methodologically.

Arafat, the second author, is a 45-year-old man from a Muslim community, born and raised in Ghent. His parents were born in Algeria and came to Belgium as labor migrants during the early 1970s. At the age of 15, Arafat began experimenting with cannabis and later tried other drugs such as cocaine and LSD, influenced by friends who did the same. By the time he was 17, Arafat started using heroin as a way to escape from the emotional burden of a home environment where he got confronted with the disappointment and anger of his parents and experienced emotional and physical abuse. Due to his substance use problems, Arafat neglected his part-time education and work trajectory (called dual learning in Belgium). He faced pressure from his school and workplace supervisors to seek outpatient treatment, where he was referred to opioid substitution treatment. Although he managed to get his life back on track for a while, his continued heroin use led his situation to deteriorate again. Eventually, Arafat ended up in a situation that can be described as ‘rock bottom’: he lost several friends to overdoses, was physically ill, lost his job, had financial issues, committed crimes, and got involved with the judicial system, resulting in a two-month prison sentence. Over the years he was admitted to short-stay crisis centers and psychiatric wards multiple times, but it was not until the age of 21, after being urged by the justice system and with the support of an imam, that Arafat’s parents agreed to his long-term admission to a residential treatment facility. An intensive care trajectory of two years in a therapeutic community enabled him to restrain from using heroin and stimulated his intrinsic motivation to stay sober. For the next two decades, Arafat remained clean, except for a six-month relapse 12 years after leaving the therapeutic community. Next to his lived experiences on the topic, Arafat has professional experience as an experiential expert in a drug-free therapeutic community (1999–2003), as an employee in an organization specifically designed for offering a continuum of care to persons with an Islamic migration background and substance use problems (El Wahda, 2004–2011) and as a trained prevention worker in schools, youth organizations, socio-cultural organizations and mosques (1999 – today). When talking about the experiences of people with an Islamic migration background in Ghent, he pours from his own experiences, but also from the many encounters he had with (families of) Muslims with substance use problems throughout his professional career.

### 2.2 Research process

Aline and Arafat had their first encounter in 2018, in which Aline discussed the topic and methodology of her PhD research with Arafat to get input from an expert-by-experience. Over the course of the next four years, they met regularly at gatherings and meetings addressing the topic of substance use recovery among persons with a migration background and ethnic minorities in Ghent. They prepared and taught a class for bachelor students at Ghent University addressing the topic of substance use and recovery among people from ethnic minorities. Looking back, preparing this class together may be considered the start of their co-creative process of meaning-making as they discovered their shared passion for fostering social change by addressing the knowledge and practice gaps regarding culturally competent substance use prevention and recovery support for persons with an Islamic migration background.

For this co-creative case study, Aline and Arafat had nine meetings in total, each lasting around two hours and spread over the course of four years. Five of the meetings took place through Microsoft Teams, two at Arafat’s home and two at the department where Aline works. During the first meetings, they discussed the personal and professional experiences of Arafat in relation to the academic knowledge of Aline and shared ideas on bottlenecks that resulted from both. Aline and Arafat also stayed in touch through e-mail, asking each other for help and keeping each other up to date on any news in the area of substance use prevention, treatment and recovery support among persons with an Islamic migration background.

In the first two meetings, Aline took the lead in starting a semi-structured interview, by asking questions about Arafat’s personal and professional experiences regarding substance use and recovery among persons with an Islamic background. From the third meeting on, Arafat started to take the lead in discussing calls for change related to his personal and professional experiences. Aline transcribed and analyzed each meeting afterwards, but it was not until the fourth meeting that Aline discussed the analyses with Arafat, facilitating further discussion and feedback. From the fourth meeting on, Aline also started to actively bring in her knowledge, based on her research experience and her analysis of the previous meetings. The meetings increasingly took the form of dialogical conversations between Aline and Arafat, delving deeper into certain themes (e.g., lived experiences of substance use problems, being an Islamic service user in a non-Islamic majority country, challenges related to the co-creative research process), exploring differences in experiences or ideas and coming to mutual understandings. At the same time, Aline started writing the manuscript with the common goal of exposing challenges and good practices regarding substance use prevention and recovery support for persons with Islamic backgrounds. This manuscript was written in Dutch, the common language between Aline and Arafat, and translated to English in the last phases of the writing process. From the fifth meeting on, the Dutch drafts of the manuscript were consistently forwarded to Arafat before the meeting and discussed during the meeting. In the last meetings, we focused on refining and finalizing the results based on the co-authors’ and reviewers’ feedback. Arafat was closely involved in the entire writing process of the manuscript, together with the other authors, who contributed to the manuscript by providing feedback and input from an academic perspective. Nevertheless, the authors recognize that this is an unfinished story, always ready to be revisited through new experiences and gained knowledge (47).

Data analysis took the shape of thematic analysis, following a three-step coding approach: open coding, axial coding and selective coding. In doing so, we followed the guidelines of Strauss and Corbin (61). After each of the initial meetings, the data was coded in an open and axial manner, while later on, the data was integrated into the mutually agreed upon framework based on the continuum of care for substance use problems through selective analysis (62, 63). The analysis was consistently revised in a co-creative and iterative manner, adding new aspects of lived, professional and academic knowledge until both parties had the feeling that saturation was reached, meaning that they had a shared understanding of what the results of this paper should comprise of and a mutual consensus that there was at the moment no need to further discuss the topics (49).

## 3 Results

The co-creative research process that aimed to identify challenges, interventions and resources regarding the continuum of care for substance use problems regarding persons with an Islamic migration background resulted in the discussion of the following themes: (1) culturally competent and selective substance use prevention, (2) facilitating access to adequate support services, (3) culturally competent substance use treatment and (4) supporting long-term recovery for persons with an Islamic background. Each section contains references to Arafat’s own experiences, his professional experiences in working with persons with an Islamic background with substance use problems, and other data (i.e., academic and grey literature, policy documents, authored articles) that was ‘plugged in’ by Aline during the conversations and the mutual development of the manuscript. The results are complemented with quotes of Arafat that are exemplary of the discussed content.

### 3.1 Culturally competent and tailored prevention

The continuum of care mainly focusses on identifying those at risk for developing substance use problems, reducing problems among those who developed a problem and increasing access to services and ongoing support (13, 14). While this entails a lot more than mere prevention initiatives that are aimed at informing people about substance use problems and opportunities for recovery, it is especially these kinds of prevention initiatives that Arafat feels are often insufficiently tailored to the needs of persons with an Islamic migration background. Therefore, this section focusses on selective prevention initiatives that aim at reducing the risks for substance use problems among persons with an Islamic migration background and increasing opportunities for early intervention and recovery (64), more specifically among youngsters with an Islamic background, Muslim communities and Muslims in prison.

In European schools, substance use prevention is often taught by teachers. In Flanders, drug prevention is under the authority of the Flemish Community Government. They have assigned the coordination of drug prevention in Flanders to VAD (Vereniging voor Alcohol en andere Drugproblemen – Center of expertise on alcohol and other drugs) (16). VAD offers training and training materials to secondary schools to provide prevention initiatives to students. However, these are not culturally tailored to ethnocultural minority students such as students with an Islamic background (65).

When Arafat was young, there were no substance use prevention initiatives in his school. Arafat was not aware of the potential dangers of heroin when he started using, allowing ignorance to turn his substance use into an addiction. It was not until Arafat was 18 and already addicted to heroin, that he felt triggered by a theater play organized by a therapeutic community. He explained that he was touched by this because he could identify with the people in the theater play, especially because one of them had an Islamic migration background.

Arafat therefor stresses the importance of culturally competent prevention campaigns and strategies, tailored to the specific needs of youngster with an Islamic background and offering recognizable role models. Hence, he believes that youngsters with an Islamic background that are at risk of developing substance use problems may benefit from receiving prevention by trained prevention workers with lived experience on both substance use problems and being a member of a Muslim minority group. When Aline brought in that scientific evidence has shown that mere testimonies of persons with lived experiences in substance use problems may not be helpful in prevention initiatives in schools (64), Arafat and Aline went further in discussion about how lived experiences can be useful in prevention of substance use problems when used and communicated carefully. The elevated understanding of the cultural sensitivities surrounding substance use problems may help prevention workers to give non-judgmental and culturally tailored prevention training to youngsters with an Islamic background (8). Also, their shared ethnocultural background may increase recognizability and relatability, important aspects in substance use prevention initiatives for youngsters (66).

*Role models are important. You’re gonna get attention. They will recognise themselves in your story. They will recognise things in your upbringing that are also important in their lives.*

In Flanders, a lot of ethnocultural minority children with fewer socio-economic resources are concentrated within certain schools (67, 68). It is precisely in these schools that children might benefit from culturally competent prevention strategies (64), as these may address culture-specific protective factors for substance use problems such as certain religious beliefs (31, 56).

According to Arafat, a lack of tailored prevention initiatives in which youngsters with an Islamic background and their families are informed about problem substance use and opportunities for care has led to a greater misunderstanding and taboo surrounding addiction among Muslim communities in Ghent.

*Tell my brothers: ‘addiction is a disease’, and they shoot me. ‘No, that is your own responsibility!’ But that's just because we're [persons with an Islamic background] behind in all that, regarding what addiction is (…). There just wasn't that much prevention going on in the community.*

This is also related to stigma concerning problem substance use in Muslim communities. Arafat describes that substance use is regarded by many Muslims as “criminal” (which, next to the aspect of substance use being ‘haram’, may be reinforced by the criminalization of drug possession), or as “derailment by the Western moral.” Stigma around substance use problems has been identified as a major barrier toward recovery resources such as an understanding network, community and access to substance use treatment (28). Additionally, cultural explanations of addiction as a cause of the “evil eye”^3^ and “spirit possession” may lead to culturally inspired help-seeking instead of mainstream treatment settings (10, 38, 69, 70). Arafat’s parents too, feeling ashamed and worried, found an explanation for Arafat’s behavior in their religion. They reached out to alternative religious healers in Algeria, but even though his faith means a lot to him, Arafat did not consider this helpful. Arafat therefore emphasizes the importance of sufficiently informing persons with an Islamic background about addiction, recovery and evidence-based interventions in a linguistically, religiously and culturally appropriate language. In turn, persons with an Islamic background can help in transforming existing prevention methods to culturally relevant ones and think about new strategies for selective prevention among Muslim minorities (71).

Between 2004 and 2011, Arafat worked in an intermediary community organization (i.e., El Wahda) which was forced to stop its activities in 2011 due to a lack of structural funding. El Wahda focused on drug prevention among ethnocultural minority groups that are less reached by mainstream prevention initiatives. They organized prevention campaigns in mosques, prisons, and schools with a high concentration of students with a migration background and low socio-economic status. Their prevention initiatives aimed to inform people with an Islamic migration background about substance use and addiction – framed from both scientific evidence and religious precepts of the Quran – and to reduce barriers to specialized drug treatment and harm reduction. In Arafat’s experiences, these interventions lowered the stigma around substance use and treatment in the community. He was frequently contacted by individuals who had attended these sessions, seeking additional information, further support from El Wahda, and referrals to services.

El Wahda also organized a project for and by mothers of children with an Islamic background, called Tuppercare (named after an international company that promotes its products at home parties), which has been put forward by the EMCDDA (European Monitoring Center for Drugs and Drug Addiction) as a good practice example of peer work for targeted prevention (72). During Tuppercare sessions, mothers are informed about substance use, addiction, and options for treatment and care in an accessible way and in their own environment, offering them a safe space to talk about their experiences in their language of choice. As Muslim communities are often considered to be family-centered, family-oriented approaches to prevention (e.g., focusing upon parent-youth dyads) have shown to be successful among Muslim minorities (71). For Arafat too, his mother was an important support and motivational figure during his entire recovery process.

*They used to compare an addict to a criminal, but suddenly they see: it’s my own son, he’s not a criminal. But also regarding treatment. Through prevention, they [people from the Muslim community] found places where they could talk about it and they realised, we are wrong to keep this topic closed and to gossip about it.*

The small crimes Arafat and his friends got involved with, gradually turned into larger drug-related criminal offences, which resulted in a 2-year prison sentence. During his time in prison, Arafat noticed an overrepresentation of persons with Islamic migration backgrounds and substance use problems. Indeed, while European research remains scarce, international research from other continents has shown that persons with a migration background and ethnic minority members with substance use problems are more likely to be convicted for crimes (including drug possession) compared to majority populations (8). The prison activities of El Wahda aimed to decrease barriers to care, by providing information about substance use problems and available services to detained persons with an Islamic background.

*Prisons are full of immigrants with an addiction. There’s overcrowding because most of them are addicted people who shouldn't be there. So do something about it; make sure that those people have time to understand what drug treatment entails. That has nothing to do with a language barrier, that is catching up because they [policy makers] have always abandoned that community (…) Because much more energy, many more resources have to go there. Instead, they’re spending less time and resources. But I don't think that's fair. That's not right. These people are Flemish people, Ghent people. These are Belgians who were born here and who need equal treatment.*

### 3.2 Facilitating access to adequate support services

Migrant and ethnic minority persons with substance use problems in Belgium are overrepresented in low-threshold, outpatient opioid substitution treatment centers, but underrepresented in residential substance use treatment services (73, 74). Additional to known cultural, language, knowledge and socio-economic barriers, this suggests an inequity in targeted referral in comparison to peers without a migration background (18, 33, 75).

Although opioid substitutes (methadone and suboxone) initially helped Arafat live a heroin-free life, Arafat simultaneously considered this dependence burdensome. When addressing this issue in substitution treatment, Arafat recalls how counselors were likely to acknowledge his successes within substitution treatment but gave him few incentives to pursue a drug-free life. Research has shown that not only persons with an Islamic migration background may face these issues, but that harm reduction – though helpful in many cases- may impede access to abstinence-based treatment for persons with substance use problems in general (76). This may, however, be even more relevant for persons with an Islamic migration background due to the additional barriers toward residential substance use treatment and mechanisms of social exclusion and referral bias (73). Arafat underscores that substitution treatment may be helpful for some, but that residential drug-free treatment should be available for people who are motivated to give it a try.

*All these minority people have not yet been to residential treatment settings, while most Flemish people have. Then they [treatment providers] say, okay, you are someone for outpatient treatment because they know in the back of their mind that residential treatment is not going to work. That is wrong reasoning. You have to do what your client needs and not what your organization can or cannot handle.*

Belgian research has reported referral bias toward more outpatient and less inpatient treatment services and service exclusion such as a longer time on waiting lists and more referral to other services after intake (73). We discussed the complexity of this matter related to language barriers and to structural inequalities, such as increased socio-economic vulnerability, but Arafat underscores that ‘cherry picking’ mechanisms of treatment providers should also not be underestimated. During his personal and professional trajectory, Arafat experienced that inpatient treatment institutions often exclude persons that they consider not ‘Western enough’ to ‘fit’ within their treatment format. This confirms the dominance of a monocultural treatment framework that may impede access to effective treatment for ethnocultural minorities such as persons with an Islamic migration background (77).

Additionally, Arafat discussed how a lack of internal motivation often serves as reason for not admitting persons with an Islamic background in substance use treatment. However, while Arafat first got pressured into treatment by family, school, work, and the judicial system, it was thanks to these admissions that he was able to grow internal motivation.

*Internal motivation usually comes when you are already in treatment. In the beginning, it's jail, and my wife is going to throw me out, I'm out of money in my account and things like that. But your real motivation comes when you see yourself grow and change.*

He therefore criticizes that internal motivation is often assessed from a monocultural framework that requires a lot of self-knowledge among service users, as well as language skills, knowledge of the treatment setting and understanding of what is perceived as internal motivation within traditional treatment culture (8). One of El Wahda’s main goals was to counteract these incorrect assumptions. They assessed motivation from a different point of view, prepared persons with an Islamic background and substance use problems for intake conversations and what to expect from treatment, and guided them to tailored care.

*People who have been in prison for a long time and at a certain point say, okay, drugs are always part of the story, I have to get help. We notice that they speak a certain street or prison language of which treatment settings think, oh no, (…) but that's a language that the service user is used to using. Those people need more time to be prepared for treatment admission.*

We discussed a case coordination meeting (Cliënten Overleg Drugs (COD) in Dutch) as a good practice regarding targeted referral to adequate support for persons with substance use problems that fall between the cracks of existing services. COD is a network of care providers who collaborate to improve continuity and coordination of care and to enhance shared decision-making in supporting persons with severe substance use problems. The aim of COD is to ensure that substance use treatment is adequately matched to service users’ profiles and needs and contributes to continuity of care (13). Additionally, case managers who advocate for service users’ needs during these COD meetings and are aware of ethnoreligious and cultural barriers, may facilitate treatment access and retention of ethnocultural and religious minority groups (8).

*You need case managers in the COD who at some point say: ‘Hey, Mohammed wants to sign up [for treatment]. Who's going to take him in?’ And then all of a sudden five, ten people, organisations are not going to keep looking at each other and saying ‘Yes, he needs treatment but I'm not going to take him in’. Then these case managers can follow up the situation of their client.*

### 3.3 Culturally competent substance use treatment

Persons with an Islamic migration background have equal rights to residential care as non-minority persons. Yet, they are less likely to find their way to residential care and are more likely to drop out (74). Therefore, it is important to look at inequity from the perspective of what can be changed from a service provider’s perspective, rather than blaming communities or individuals in recovery (18, 24, 78).

Arafat underscores how substance use treatment is still very monocultural. His religion played a crucial role in his recovery and personal life. His connection with Allah and his commitment to remain loyal provided motivation during difficult times. Being part of an ethnoreligious community also contributed positively to his sense of identity, belonging, and connectedness. However, during treatment, his religious identity was not always acknowledged, leading him to advocate for his rights and collaborate with others from an Islamic background to address issues like access to halal food and time for prayer.

Although there is a significant amount of heterogeneity in how Muslims experience and practice their faith, Arafat emphasizes the importance of residential substance use treatment facilities providing opportunities for individuals to practice their faith. This may include facilitating access to halal food, enabling participation in Ramadan, and providing adequate time and space for prayer. Given the significance of these religious practices within the Muslim faith, they must be made available and accommodated within a treatment setting. However, Arafat acknowledges that aligning Islamic and treatment prescriptions may come with some challenges. Collaboration between treatment providers, persons with knowledge of Islam (e.g., intercultural mediators) and service users is essential to find compromises between treatment and service user cultures (79). This facilitates the integration of mainstream and ethnoreligious models of explanation, support and treatment, taking into account service users’ needs and contexts. In this regard, Arafat recounts a specific situation in which he engaged a teacher of Islam to explore how a service user with psychosis could fast during the holy month of Ramadan without endangering his treatment trajectory.

*Do you want to fast in the month Ramadan? You don’t need to, but I understand that you want it, because it’s important for your recovery. So take your medication but also do the fasting.” That way we found a compromise.*

By insufficiently supporting religion and culture as recovery resources and important aspects of their identity, substance use treatment may contribute to a “Westernization” or deculturation of persons with an Islamic background in treatment, which, in turn, may lead to cultural bereavement and a decrease of religious and cultural recovery resources (31, 79). While Arafat acknowledges that inpatient treatment settings have undergone an evolution toward more inclusive treatment since he was a service user from 1994–1999 and 2011–2012, for example in providing halal food, barriers to and in care for people with an Islamic background are still relevant (80). To facilitate long-term recovery, Arafat beliefs that treatment settings should support service users to build religiously and culturally relevant recovery resources.

*If your [referring to service providers] objective is to provide equal counselling, if the [treatment] organization believes that group discussions are important, that family discussions are important to mend the bond, that finding meaning is important, values and norms, and this person’s [service user] values and norms is their faith, then you are going to look for those things in your organization: can I offer them that and how? (…). But to transform this person into a ‘Belgian’, to really say, we're not going to adapt, and then after two years these persons must go back into the community and then they are completely lost and haven’t been doing anything regarding their faith or community at all. Then we [service providers] are doing it wrong.*

In Arafat’s experiences, available therapies to process and cope with experiences in the past, which are considered an important aspect of substance use recovery (81), are insufficiently adapted to the needs of persons with an Islamic migration background.

*You see that there are certain aspects of therapy that you can’t make use of because it’s not adapted. Then there is the question: should I be the one adapting or the other? I speak Dutch, but I can’t help it that my origin is different and that certain aspects will have to be dealt with differently during psychotherapy compared to a typical Flemish person.*

Therapists with a similar ethnoreligious background and language may therefore be more suited for offering psychotherapy to persons with an Islamic background (22, 24). However, we discussed that the profiles of substance use treatment providers and counselors hardly reflect Flanders’ super-diverse society, let alone the superdiverse population of persons with substance use problems (80).

The involvement of a recovery-supportive social network in substance use treatment has proven to be of major importance for recovery, also for Arafat: as a source of motivation, as support and as a continuous presence in his life (31, 82). However, involving the network of persons with an Islamic background in substance use treatment may require some extra effort, given barriers such as language, the stigma surrounding substance use and treatment, distrust toward mainstream health care and practical barriers due to socio-economic reasons (18, 83).

Treatment that insufficiently answers to the needs of persons with an Islamic background in terms of faith, culture, family work and psychotherapy can cause drop-out (84). In Arafat’s experience, however, drop out is in such cases often attributed to the individual and/or their ‘culture’, rather than the provision of care and its ability to adapt to that person’s needs.

*The coordinator said: ‘We’re in Flanders, you know’. Once we’re talking about interculturality, they hit the brakes. But why? Look around in your city. Society is becoming this way. You will have to change. And if he [person with an Islamic background] feels that the coordinator doesn’t want to help and understand him, he takes his bags and leaves. And then it’s easy for treatment providers to say: it was his choice to leave.*

To ensure equitable substance use treatment, we agreed that a structural and critical view on the access to and drop out of persons with an Islamic background in substance use treatment, as well as how substance use treatment is organized and subsidized in light of the superdiverse society, is needed.

*Our society is changing. We should adapt to that society and look for solutions on how to support them [people with an Islamic background and substance use problems] better. (…) There are going to be people saying ‘Are these people still not integrated?’ No, they are integrated, but they are not getting the right substance use treatment, or equitable substance use treatment. (…) Subsidies [for substance use treatment programs] should be much more result oriented, based on the percentage of successful treatment completion among ethnocultural minorities.*

According to Arafat, the discussion about ethnocultural diversity in substance use treatment and policy is still too often dominated by what he calls ‘alibis’: invalid excuses to argue why persons with an Islamic background are insufficiently reached (e.g., language barriers, while a lot of persons with an Islamic background in Flanders speak Dutch), but also alibis to ‘prove’ diversity such as, for example, repeatedly referring to the involvement of a Muslim in policy-making without structurally focusing on multicultural diversity in multiple areas. We discussed how – while Muslim representatives in treatment and policy matter – this kind of tokenism may have a major impact on both the wellbeing of those who are used as tokens, challenging their ongoing engagement in a field that is already dominated by majority group members and the communities in question, who may be left out of the conversation because of this one token (42, 85).

### 3.4 Supporting long-term recovery

Depending on the conceptualization of recovery, relapse has been defined as (a) an interruption of abstinence common to addiction, (b) a vulnerability to uncontrollable substance-related behavior and/or cues and (c) a transition to potential progression or regression. To minimize relapse and its negative consequences (regardless of the definition of relapse used), relapse prevention and relapse-sensitive care are essential (86).

Arafat encountered a relapse 12 years after leaving the therapeutic community, initiated by the combined pressure of his job, life adversities, lack of professional support and space to show emotional vulnerability, as well as the temptation to believe that one shot of heroin would not harm his recovery. We discussed that relapse is part of many recovery processes and that treatment settings should also approach it that way (77). Arafat believes that treatment settings should engage even more in supporting the recovery process of persons who encountered a relapse, by enabling connection, therapeutic relationships and offering new chances. In his experience, many people get into worse shapes after a relapse, due to exclusion processes of residential treatment settings, a loss of support after a person is considered ‘recovered’ and long waiting lists at treatment centers. This may impact persons with an Islamic background even more, due to additional structural and social barriers toward recovery resources and support (31, 40), increasing the importance of outpatient follow-up and collaboration with social network and support systems (87). In his personal story, Arafat considers himself lucky that he was able to get into residential treatment soon after his relapse. This enabled him to regain control over his life and refrain from heroin within six months after his first re-use of heroin.

The conversations of Aline and Arafat touched upon the importance of continuity of care in multiple ways, both in drug treatment and post-treatment follow-up, between various support services concerning different life domains, and between professional and informal support. Arafat refers to El Wahda as a good practice of continuity between different services and support sources, before, during treatment and post-treatment. Besides their role as cultural mediators, they conducted case management, followed up care trajectories, informed and involved families in the recovery process of their close ones and brought together networks of formal and informal, traditional and ethnocultural forms of care.

*At some point there is so much care that no one knows who is doing what, but then there is one person saying: you are the case manager and you follow up on the whole situation. And this case manager brings the social workers together regularly. Are we going in the right direction? What is going well? What is not? Is it about employment? Is it financial? (…) Who is working on what? And make agreements in that direction. (…) And then you also feel that the organisations who want to take them [persons with an Islamic background] in but hesitate and think: ‘what about that family?’, then we [El Wahda] say: then you can count on us. And then we will support them. And for some we include the imam and for others we include an intercultural mediator, and for others, whatever.*

Increased continuity of care can also facilitate a smooth transition from residential treatment settings to home environments (88). We agreed on the responsibility of substance use treatment to collaborate with social and community networks in order to facilitate the transition process after treatment, as well as on the importance of stigma-decreasing initiatives in religious communities and society to build recovery-supportive environments (28, 79, 83).

Besides professional support and substance use treatment, meaningful daytime activities in the form of a job, voluntary work or education, as well as physical recovery capital such as housing and financial stability have proven important for recovery (17). However, persons with an Islamic background in recovery of substance use problems may encounter additional difficulties building these kinds of resources because of stigma and discrimination (28). For example, Arafat encountered discriminatory experiences regarding housing related to his name which clearly suggests an Islamic/Arabic migration background. Furthermore, issues of stigma regarding a criminal record in relation to a history of substance use problems made it difficult for him to find a job other than drug counseling.

*Employers don't want to take a risk because suppose someone finds out you have a criminal record, then that director will be in the news or whatever. And then they're scared and then that's why they're not going to invite you or say you're chosen, whereas that's just me wanting to get out of that addiction business and do something new for once, but yeah…*

Hence, addressing multiple stigma on macro (societal) and meso (community) level is essential to facilitate long-term recovery among persons with an Islamic migration background in a non-Islamic majority country (28).

## 4 Discussion

This paper is the result of a co-creative research process between an expert-by-experience and an academic researcher ‘plugging in’ her academic knowledge. In doing so, we actively and dynamically bring together data and theory, insider and outsider perspectives, theory and practice (47, 89). The topics of substance use prevention, facilitating access to adequate recovery support services, culturally competent substance use treatment and supporting long-term recovery through relapse-sensitive care, aftercare, continuity of care and recovery capital are discussed. Each theme delves into the specific situation of persons with an Islamic background in Flanders (Belgium) as a group of people, albeit very heterogeneous, that is often overlooked. The study shows that persons with an Islamic migration background may face several barriers to effective substance use prevention and treatment across the continuum of care and gives an indication of what may be done to overcome these barriers. This discussion concludes with some critical reflections.

In the study, we discuss the experienced ‘good practice’ of an intermediary community organization by and for persons with an Islamic migration background that facilitated targeted substance use prevention and recovery support in Ghent (El Wahda). This intermediary body also provided case management to increase continuity of care and to bridge the gaps between what support was available and what support was needed. While such an organization can offer hands-on (though band-aid) solutions for persons with an Islamic background who are considered ‘hard-to-reach’ in the current landscape of substance use prevention and recovery support, the fact remains that such organizations bridging the gaps should not be necessary in a well-functioning treatment system. Hence, it remains the responsibility of substance use prevention and care services to critically examine why the dominant treatment services fail to reach this group (24, 42, 90). Cultural competence, defined as “the process in which the healthcare provider continuously strives to achieve the ability to effectively work within the cultural context of a service user (individual, family or community)” (91), p. 203, as well as systemic solutions for health inequities, should ideally obviate intermediate organizations advocating for those who are now disadvantaged (24, 92). Furthermore, recovery-oriented systems of care, integrating different formal and informal, direct and indirect care and support systems, should facilitate person-centered care that is both culturally competent and continuous (15, 31, 79).

Throughout the study, it becomes clear that the continuum of care may be insufficiently adapted to cultural aspects and expectations of substance use prevention and care among Islamic minority communities (18). Bypassing cultural sensitivities, needs and strengths in substance use treatment violates the universal human right of each individual to participate in cultural life without any distinction regarding race, language or religion (24, 40, 90). It may lead to cultural bereavement and loss of ethnocultural identity and resources (79). By enhancing sensitivity for differences in world views, cultural traditions and upbringing, culturally competent prevention and care strategies may increase socio-cultural relevance for service users, while the effectiveness in terms of prevention outcomes is still understudied (64, 93).

However, prevention and recovery support specifically targeted to people with an Islamic background may be equally exclusionary, as they hold the danger of essentializing, othering and overlooking other intersectional traits of diversity, such as age, gender, sexual orientation, language, religiousness, social class, migration status and local embedding (24, 90, 94). Furthermore, in uncovering and addressing the strengths and needs of persons with an Islamic background, there is a thin line between ethnocultural awareness and stereotyping, between empowerment and disempowerment, between cultural competence and culturalization (i.e., the misinterpretation of social inequities as cultural differences) (3, 89, 95). Even more so, De Kock warns that even the cultural competence movement risks reinforcing a dominant neoliberal and biomedical discourse on what constitutes ‘good treatment’ by allocating the source of the ‘problem’ within service users’ cultural background (96). More research is needed to develop prevention initiatives and recovery support that are both evidence-based and specific enough, not resulting in unnecessary othering but with respect for group and individual-level needs (7). In particular, since the omission of migrants and ethnic minorities in policy-making processes has significantly contributed to inequalities at the level of treatment services (80), the active inclusion of minority-group members in this critical analysis and in the creation of novel interventions is essential (71).

This study is not an objective and generalizable representation of facts, nor is it a subjective qualitative analysis by a researcher. It does not convey to depict a truth that is generally difficult to grasp within human experiences. It focusses on the experiences of a single case, but brings these micro-level experiences to social theorizing through the process of problem posing, dialog, and reflection. The lived experiences of Arafat and stories of other Muslims he got in contact with during his professional career, linked to the broader context of academic literature, show that these experiences do not exist in a vacuum and can teach us about how macro-level mechanisms come to life in micro-level and meso-level settings, as well as what meso- and micro-level answers are sought for macro-level problems (18, 97). This required a search for a common language, continuously aiming for balance between academic requirements and language on the one hand, and staying close to the authentic expressions, experiences and expectations of Arafat on the other (92). By doing so, academic, practice-based and real-life-based knowledge becomes more than the sum of its parts (89). As Davies (98) puts it: “Ideas, concepts and practices like particles of light, ripples on a pond, or crisscrossing waves on the ocean, affect each other. They interfere with each other. Ideas and matter similarly affect each other” (p. 4).

Nevertheless, several limitations also need to be acknowledged. First, by exploring the continuum of care for substance use problems regarding persons with an Islamic migration background, we were able to uncover how the different aspects of this continuum interrelate with each other and how an intervention on one aspect of the continuum might positively affect other aspects of the continuum as well. For example, committing to tailored substance use prevention in Islamic communities may decrease barriers to care and increase the number of cultural recovery resources after care. However, it is important to acknowledge that the broad scope of the study may impede its ability to provide sufficient detail. Consequently, certain aspects within the continuum may not receive comprehensive coverage, potentially limiting the depth and breadth of the findings. Second, the lived and professional experiences included in this study arise from one single person. To increase the validity of the statements in this manuscript, more research is needed to compare these experiences to other personal and professional experiences across a diversity of persons with an Islamic migration background. More research on a larger scale is needed to identify mechanisms of exclusion of persons with an Islamic migration background on the continuum of care for substance use problems and provide an incentive to further develop tailored prevention initiatives and recovery support for persons with an Islamic migration background. Third, a lack of research focused on persons with an Islamic migration background as a religious minority, obliged us to include studies discussing the broad and heterogeneous group of persons with a migration background and ethnic minorities. While persons with an Islamic migration background are part of this population, more research is needed to uncover specific aspects related to having an Islamic migration background. Last, while incorporating academic knowledge in the co-creative research process has its advantages to come to a multilayered understanding of the phenomenon, it may also increase confirmation of what already exists, rather than truly centralizing lived experiences (45). Therefore, we further encourage bringing marginalized voices of persons with lived experiences to the fore in research, policy and practice, with a critical self-analysis of how to represent these voices and collaborate with their owners (51, 83).

## Data availability statement

The datasets presented in this article are not readily available because of ethical and privacy reasons. Requests to access the datasets should be directed to aline.pouille@ugent.be.

## Ethics statement

The studies involving humans were approved by Ethics Committee of the Faculty of Psychology and Educational Sciences of Ghent University. The studies were conducted in accordance with the local legislation and institutional requirements. The participants provided their written informed consent to participate in this study. Written informed consent was obtained from the individual(s) for the publication of any potentially identifiable images or data included in this article.

## Author contributions

AP and AB: conceptualization, study design, methodology, data-collection, and data-analysis. AP, AB, CR, FV, and WV: writing manuscript and review and editing. FV and WV: supervision, project administration, and funding acquisition. All authors contributed to manuscript revision, read, and approved the submitted version.

## Footnotes

^1^In this study we discuss the continuum of care for substance use problems regarding the heterogeneous group of persons with an Islamic migration background in Flanders (Belgium). This includes first-generation migrants (newly arrived or settled migrants) who originate from Islamic-majority countries and self-identify as Muslim, but also further-generation diasporic Muslims that are an ethnoreligious and – since we approach religion as a form of culture - an ethnocultural minority in Belgium (3, 4). Since this study takes a meta-perspective on the needs of ‘persons with an Islamic migration background’, this may lead to some statements that seem generalizing. Nevertheless, readers should always take into account that many intersecting identities influence persons’ experiences and the ‘group’ of persons with an Islamic migration background, does not exist in a homogeneous manner.

^2^This study approaches substance use recovery or recovery from problem substance use from the perspective of what is called ‘the new recovery paradigm’, which defines recovery as “a dynamic, personal, and multidimensional process of change, characterized by increased wellbeing on multiple life domains” (11), p. 2.

^3^The evil eye is a look or gaze of resentment or jealousy that can cause harm or misfortune to the person it is directed towards (56,57).
